# Preoperative endoscopic tattooing to mark the tumour site does not improve lymph node retrieval in colorectal cancer: a retrospective cohort study

**DOI:** 10.1186/s12952-015-0027-7

**Published:** 2015-05-07

**Authors:** Carlo V Feo, Mattia Portinari, Michele Zuolo, Simone Targa, Vincenzo G Matarese, Roberta Gafà, Elena Forini, Giovanni Lanza

**Affiliations:** Department of Surgery, Unit of Clinica Chirurgica, S. Anna University Hospital of Ferrara, and University of Ferrara, Via Aldo Moro, 8 Room 2 34 03 (1C2), 44124 Ferrara, Cona, Italy; Department of Medicine, Unit of Gastroenterology, S. Anna University Hospital of Ferrara, Ferrara, Italy; Department of Diagnostic Imaging and Laboratory Medicine, Unit of Anatomic Pathology, S. Anna University Hospital of Ferrara, and University of Ferrara, Ferrara, Italy; Unit of Statistics, S. Anna University Hospital of Ferrara, Ferrara, Italy

## Abstract

**Background:**

A direct correlation between number of lymph nodes retrieved and evaluated after a colectomy for colorectal cancer and survival of the patient has been reported, and consensus guidelines recommend to assess at least 12 lymph nodes for adequate staging. Many factors (i.e., patients’ and tumour characteristics, surgeon, and pathologist) may influence the evaluation of the presence of neoplastic disease in lymph nodes as well as the total number of lymph nodes examined. Preoperative endoscopic tattooing to mark the site of the tumour has recently been suggested to facilitate the retrieval of lymph nodes in colorectal specimens. The aim of this study was to investigate its association with adequate lymphadenectomy (≥12 nodes) after colorectal resection for cancer.

**Results:**

All patients undergoing elective colorectal resection for cancer between 2009 and 2011 at the S. Anna University Hospital in Ferrara, Italy (N = 250) were retrospectively divided into two cohorts according to whether ink tattooing to mark the tumour site was performed during preoperative colonoscopy. The two cohorts were comparable regarding age, gender, body mass index, tumour location and size, TNM staging, and DNA microsatellite instability-high status. No difference between the tattoo (N = 107) and control (N = 143) groups could be detected in the rate of adequate lymphadenectomies performed (78% vs. 79%, p = 0.40). All factors known to influence lymph nodes retrieval from colorectal specimen were specifically evaluated. Rectal and colonic cancers were analysed together and separately. Full adjusted logistic regression analysis in patients who underwent colonic resection showed that right hemicolectomy (OR 4.72; CI95% 1.09-20.36) was the only factor associated to adequate lymphadenectomy. No association between ink tattooing performed preoperatively to mark the site of the tumour and adequate lymphadenectomy after colorectal resection was found with logistic regression analysis.

**Conclusion:**

This study shows that preoperative ink tattooing utilized to mark the site of the tumour does not improve adequate lymphadenectomy and lymph nodes yield from colorectal cancer specimens. Further studies are therefore needed to determine if preoperative colonoscopic tattooing to mark the tumour site can refine staging.

## Background

In Italy, colorectal cancer is the second most reported malignancy in women and the third among men and is the second leading cause of cancer-related death in both men and women [1]. A surgical resection is the only curative modality for localized colon cancer and its goal is to remove the tumour as well as the lymph nodes draining the affected segment (regional lymphadenectomy) to provide important prognostic information, and guide postoperative treatment. Also, a direct correlation between number of lymph nodes retrieved and evaluated after a colectomy and survival of the patient has been reported [2]. Consensus guidelines recommend to assess at least 12 lymph nodes for adequate staging [3] and to consider the use of postoperative chemotherapy for patients with node-negative disease and less than 12 nodes in the surgical specimen [4]. Many factors may affect the presence of the disease in the lymph nodes as well as the total number of lymph nodes examined, including surgical technique and the methodology utilized for evaluation of the nodes. For instance, complete meso-colic resection and central venous ligation could potentially yield more lymph nodes by removing more tissue around the tumour [5] while being diligent in the search and utilizing measures such as “fat clearing” can improve the detection of nodes during pathology examination. Endoscopic preoperative ink tattooing *to mark the site of the tumour* has recently been suggested to facilitate the retrieval of lymph nodes in colorectal specimens [6,7]. The aim of this study was to investigate the association between ink tattooing performed preoperatively *to mark the site of the tumour* and lymph nodes retrieval by the pathologist after colorectal resection for cancer.

## Results

Two-hundred and fifty patients were enrolled for this study; 193 had a colonic tumour and 57 a rectal tumour. The demographic data are illustrated in Table 1.

**Table 1:** **Demographic data and BMI in the patients with colonic and rectal tumours**

	**Tattooed group N = 107**	**Control group N = 143**	**P**
**Age (years)**	71.3 (62.4-78.0)	73.3 (65.9-80.0)	0.14
Colonic cancer	71.1 (62.1-77.7)	74.3 (67.2-80.5)	0.05
Rectal cancer	74.5 (65.9-79.9)	72.4 (63.6-79.2)	0.50
**Gender**			
**(Male/Female)**	50 (47%)/57 (53%)	82 (57%)/61 (43%)	0.09
Colonic cancer	45 (46%)/52 (54%)	52 (54%)/44 (46%)	0.20
Rectal cancer	5 (50%)/5 (50%)	30 (64%)/17 (36%)	0.40
**BMI** ^a^	26.0 ± 3.5	24.7 ± 4.0	0.08
Colonic cancer	25.8 ± 3.5	24.7 ± 3.9	0.20
Rectal cancer	28.5 ± 2.6	24.6 ± 4.1	0.08

^a^The BMI was available only for the 97 patients who were operated on in the year 2011, 46 (4 with rectal cancer) in the tattooed group and 51 (20 with rectal cancer) in the control group.

One hundred and ninety-eight (79.2%) procedures were performed using an open approach while 52 (20.8%) were performed laparoscopically; of these, 19 were converted to an open laparotomy.

The tumour-specific characteristics and nodal staging are illustrated in Tables 2 and 3, respectively.

**Table 2:** **Tumour-specific characteristics in patients with colonic and rectal tumours**

	**Tattooed group N = 107**	**Control group N = 143**	**P**
**Location in the colon**	97	96	
Right colon	40 (41%)	52 (54%)	0.09
Tansverse colon	1 (1%)	3 (3%)	--
Left colon	56 (58%)	41 (43%)	0.05
**Location in the rectum**	10	47	<0.01
**Tumour size (cm)**	4 (3–5.9)	4.7 (3.25-6)	0.08
Colonic cancer	4.5 (3–6)	5 (3.5-6.5)	0.04
Rectal cancer	3.5 (2.5-3.75)	4 (2.75-6)	0.18
**pT Stage in colon cancers** ^a^			0.12
Tis	1 (1%)	2 (2%)	
T1	7 (8%)	2 (2%)	
T2	12 (13%)	5 (6%)	
T3	48 (52%)	54 (61%)	
T4	24 (26%)	25 (29%)	
**pT Stage in rectal cancers**			0.18
Tis	0	0	
T1	0	4 (9%)	
T2	5 (50%)	7 (15%)	
T3	3 (30%)	28 (59%)	
T4	2 (20%)	8 (17%)	
**DNA Microsatellite Instability-High**	10/97 (10%)	10/96 (10%)	--

^a^In 13 patients final pathology revealed an adenoma with high grade dysplasia.

**Table 3:** **Staging of the lymph-nodes**

	**Tattooed group N = 107**	**Control group N = 143**	**P**
**N of lymph nodes examined**	16.0 (12–24)	17.0 (12–21)	0.90
**N of nodes examined in colonic cancer**	17.0 (12–24)	18.0 (13–23.75)	0.40
Right colon	21.0 (14–28)	19.0 (15.25-26)	
Tansverse colon	14.0^a^	15.0 (9–19)	
Left colon	14.5 (11–21.5)	16.0 (12–20)	
**N of nodes examined in rectal cancer**	12.0 (9.75-28.25)	13.0 (10.75-20)	0.70
**pN stage in colon cancers** ^b^			0.70
0	50 (55%)	50 (58%)	
1	23 (25%)	19 (22%)	
2	18 (20%)	17 (20%)	
**pN stage in rectal cancers**			0.60
0	5 (50%)	25 (53%)	
1	4 (40%)	13 (28%)	
2	1 (10%)	9 (19%)	
**N adequate lymphadenectomy (≥12)**	84 (78%)	113 (79%)	
Colonic cancer	78/97 (80%)	80/96 (83%)	0.40
Rectal cancer	6/10 (60%)	33/47 (71%)	0.30

^a^Only one tattooed patient had a tumour centrally located in the transverse colon and underwent a transverse colectomy.

^b^In 13 patients final pathology revealed an adenoma with high or moderate dysplasia.

Two patients (7%) out of a random sample of 30 patients in the tattoo group showed the presence on the pathology slides of microscopic ink.

Eleven surgeons performed the 250 colorectal resections: four surgeons contributed 203 cases, one surgeon 12 cases, while the other six had 5 or fewer cases. Ninety-five operations (89%) in the tattoo group and 125 (87%) in the control group were performed by “high-volume” surgeons. The number of lymph nodes retrieved in colorectal specimens from patients operated on by high-volume and low-volume surgeons was 17.0 (IQR, 12–23) versus 15.5 (IQR, 13.5-24.25), respectively (P = 0.8).

Twelve pathologists examined the surgical specimens and two of them had specialized expertise in colorectal diseases. These two pathologists examined 43 cases (40%) in the tattoo group and 65 (45.5%) in the control group compared to 64 (60%) and 78 (54.5%), respectively performed by a non-dedicated pathologist. Dedicated pathologists retrieved 18.0 (IQR, 12–25) lymph nodes in the tattoo group compared to 18.0 (IQR, 13.5-26) in the control group (P = 0.3), while non-dedicated pathologists retrieved 15.0 (IQR, 12–23) lymph nodes and 15.0 (IQR, 11–19) lymph nodes, respectively (P = 0.3). The number of lymph nodes retrieved by dedicated and non-dedicated pathologists was 18.0 (IQR, 13–24.75) versus 15.0 (IQR, 11–20), respectively (P = 0.001). The distribution of the patients among high- versus low-volume surgeons and dedicated versus non-dedicated pathologists is illustrated in Table 4.

**Table 4:** **Distribution of patients with colonic and rectal cancer among high- versus low-volume surgeons and dedicated versus non-dedicated pathologists**

	**Dedicated pathologists N = 108**	**Non-dedicated pathologists N = 142**
**High-volume surgeons**		
N = 220	95 (43%)	125 (57%)
**Low-volume surgeons**		
N = 30	13 (43%)	17 (57%)

Unadjusted logistic regression including all patients (N = 250) showed that dedicated pathologists (OR 1.97, 95% CI 1.03-3.78) and colon resection (OR 1.97, 95% CI 1.00-3.87) were significantly associated with adequate lymphadenectomy (Table 5), but these associations were not confirmed after adjusting for potential confounders. Unadjusted logistic regression analysis in patients who underwent left or right colorectal resection (N = 189) was also performed and showed that right colonic resections were significantly associated with adequate lymphadenectomy (OR 2.70, 95% CI 1.21-6.01). This association was maintained after adjusting for potential confounders (Table 6). The association between ink tattooing performed preoperatively to mark the site of the tumour and lymph nodes retrieval after colorectal resection was not found with logistic regression analysis (Tables 5 and 6).

**Table 5:** **Association between baseline characteristics and adequate lymphadenectomy according to logistic regression models adjusted for potential confounders, in all patients undergoing surgical resection of the colon or rectum for cancer (N = 250)**

	**Adequate lymphadenectomy (≥12)**			
	**Unadjusted model**		**Full adjusted model**	
**Characteristics**	**Odds ratio (95% CI)**	**P**	**Odds ratio (95% CI)**	**P**
**Gender (male)**	1.17 (0.63-2.15)	0.625	1.94 (0.69-5.46)	0.207
**Age** ^a^	1.00 (0.98-1.03)	0.781	0.99 (0.95-1.05)	0.924
**BMI** ^a^	0.91 (0.81-1.03)	0.139	0.90 (0.80-1.02)	0.112
**Pathologists (dedicated)**	1.97 (1.03-3.78)	0.041	2.96 (0.80-10.94)	0.105
**Surgeons (high-volume)**	0.55 (0.18-1.65)	0.284	0.27 (0.03-2.48)	0.249
**Type of resection (colon)** ^b^	1.97 (1.00-3.87)	0.050	3.02 (0.94-9.69)	0.063
**Surgical technique (laparotomy)**	1.25 (0.53-2.96)	0.611	1.52 (0.44-5.33)	0.511
**Preoperative colorectal tattooing (yes)**	0.94 (0.51-1.74)	0.837	0.94 (0.32-2.75)	0.915

^a^Odds Ratio for age and BMI refer to increasing age and increasing BMI, respectively.

^b^Reference category was patients with resection for rectal cancer.

**Table 6:** **Association between baseline characteristics and adequate lymphadenectomy according to logistic regression models adjusted for potential confounders, in patients affected by cancer undergoing surgical resection of the left or right colon (N = 189)**

	**Adequate lymphadenectomy (≥12)**			
	**Unadjusted model**		**Full adjusted model**	
**Characteristics**	**Odds ratio (95% CI)**	**P**	**Odds ratio (95% CI)**	**P**
**Gender (male)**	0.86 (0.41-1.80)	0.680	0.99 (0.28-3.57)	0.997
**Age** ^a^	0.99 (0.96-1.03)	0.764	0.99 (0.93-1.06)	0.893
**BMI** ^a^	0.91 (0.79-1.06)	0.214	0.88 (0.75-1.04)	0.142
**Pathologists (dedicated)**	1.63 (0.76-3.53)	0.213	1.51 (0.31-7.23)	0.607
**Surgeons (high-volume)**	0.65 (0.18-2.34)	0.513	0.76 (0.07-8.51)	0.824
**Type of resection (right colon)** ^b^	2.70 (1.21-6.01)	0.015	4.72 (1.09-20.36)	0.038
**Surgical technique (laparotomy)**	1.68 (0.68-4.15)	0.261	3.85 (0.87-17.10)	0.077
**Preoperative colorectal tattooingv**	0.78 (0.37-1.64)	0.513	1.09 (0.30-3.95)	0.900

^a^Odds Ratio for age and BMI refer to increasing age and increasing BMI, respectively.

^b^Reference category was patients with resection of the left colon.

## Discussion

This retrospective study shows that performing ink tattooing during preoperative colonoscopy to mark the site of the tumour does not increase the rate of adequate lymph node analysis in colorectal cancer specimens.

Identifying the spread of the neoplastic disease to the lymph nodes is of the utmost importance to determine both the prognosis and the optimal treatment in patients with colorectal cancer [8,9]. Recently, the use of ink tattooing to mark the tumour site during preoperative colonoscopy has been reported to enhance the quality of the pathology examination by increasing the number of lymph nodes retrieved and of adequate lymphadenectomy in colorectal cancer specimens [6,7]. Many factors, however, may influence the evaluation of the presence of neoplastic disease in lymph nodes as well as the total number of lymph nodes examined. These factors were all taken into account in this study and include patients’ and tumour characteristics, surgeon, and pathologist [10].

Older patients with colorectal cancer seem to have fewer lymph nodes retrieved in specimens possibly because nodes number and size is reduced by the aging process or segmental colectomies are performed due to more comorbidities compared to younger patients [11,12]. The relationship between female gender and lymph nodes yield in colorectal cancer is controversial: a few studies suggest an increased number of nodes in women [13,14], but the majority of authors did not find a correlation [11,15-17]. An inverse relationship has been suggested between number of lymph nodes yielded and patients’ BMI [11], although not confirmed by other investigators [18]. In our study, the two groups were well balanced with respect to age, gender, and BMI, even though among patients with rectal cancer a prevalence of women (50% vs. 36%, P = 0.4) and higher BMI (28.5 vs. 24.6, P = 0.08) in the tattoo versus control group was observed (Table 1). Full adjusted logistic regression analysis did not reveal an association between age, BMI, and gender with adequate lymphadenectomy (Tables 5 and 6).

The specific characteristics of the tumour are also important elements to be considered. Tumours situated in the rectum tend to yield fewer lymph nodes than colonic tumours [19]. Accordingly, the number of lymph nodes retrieved and adequate lymphadenectomies performed in this series was lower in patients with rectal tumours compared to colonic tumours (Table 3). Full adjusted logistic regression analysis, however, did not confirm an association between adequate lymphadenectomy and type (i.e., colonic versus rectal) of resection (Table 5).

As far as preoperative colorectal tattooing is concerned, to ink-mark the site of the tumour was not associated to adequate lymphadenectomy after adjusting for potential confounders (Tables 5 and 6).

Tumours located in the right colon have been reported to yield more lymph nodes compared to sigmoid, recto-sigmoid, and rectal tumours [11]. In part that could be due to the more extensive mesenteric resection performed during right hemicolectomies. Accordingly, unadjusted logistic regression analysis in this study showed that right colonic resections were significantly associated to adequate lymphadenectomy (OR 2.70, 95% CI 1.21-6.01), also after adjusting for potential confounders (Table 6). The number of right colectomies performed in the tattoo group was less than in control group (41% vs. 54%, P = 0.09), possibly because the ileocecal valve represents an endoscopic landmark and surgical resections are more standardized. As a matter of fact, preoperative endoscopic tattooing is generally performed to allow or facilitate intraoperative localization of colorectal tumours that cannot be detected by inspection during laparoscopic resections or manual palpation during open resections (e.g., early lesions, resected polyps). Tumour size [11,20] and advanced pT stage [21,22] have both been related to higher lymph nodes yield in colorectal cancer and again, there was no difference between groups for these variables in our series (Table 2). More recently, a DNA MSI-H status has been reported to be associated with higher lymph node counts, which may represent an active immune response to high amounts of neoantigens in tumours with such a phenotype [23,24]. MSI-H status, however, is more frequent in right-sided colorectal cancer, which has higher lymph nodes yields compared to left-sided lesions as previously noted. Nonetheless, there was no difference in the MSI-H status between the tattoo and control groups in our series (10% vs. 10%).

The analysis of the distribution of patients among surgeons with high- versus low-volume and pathologists with specialized expertise in colorectal diseases versus others revealed no difference (P = 0.98) (Table 4). The surgeon case volume was not associated to an increased lymph node yield or number of adequate lymphadenectomies (Tables 5 and 6), which is in conflict with previous reports [25]. By contrast, pathologists dedicated to colorectal diseases retrieved a higher number of nodes compared to non-dedicated pathologists (18.0 vs. 15.0, P < 0.001), but the lymph node yield did not differ in both the tattoo and control groups for dedicated (18.0 vs. 18.0, P = 0.3) and non-dedicated pathologists (15.0 vs. 15.0, P = 0.3). These data seem to confirm that the pathologist, rather than preoperative tattooing, is an important element determining the number of lymph nodes retrieved and analysed [25]. However, full adjusted logistic regression analysis including all patients (N = 250) did not find an association between adequate lymphadenectomy and dedicated pathologists (Table 5).

In stark contrast with this investigation, other Authors have reported an increase in number of lymph node yield in colorectal cancer specimen with preoperative ink tattooing of the tumour site [6,7]. Dawson and collaborators, performed a retrospective study to assess the adequacy of lymph node analysis (≥12 nodes) with respect to ink tattooing during preoperative colonoscopy in 174 patients with colonic cancers (62 tattooed) and 35 with rectal cancers (8 tattooed) [6]. The median number of lymph nodes examined in tattooed and non-tattooed specimens were 19 (range, 7–77) and 16 (range, 2–74), respectively whereas there was a 15% absolute difference in the adequacy of node analysis (87% vs. 72%) in favour of tattooed patients. Unfortunately, however, no evaluation of potential confounders was performed. The Authors concluded advocating routine tattooing of all suspicious neoplasms at the time of preoperative colonoscopy, as it seemed to increase the quality of lymph node analysis [6]. Of note, they reported 46% right colectomies among the 62 tattooed patients with colonic cancer versus 70% in 112 non-tattooed patients. Therefore, it is unlikely that less right colectomies in the tattoo group versus controls in our series of patients (41% vs. 54%, P = 0.09) has influenced our results.

Subsequently, Bartels et al. performed a retrospective case–control study defining tattoo patients by presence of microscopic carbon dye lymph nodes deposits in the pathology reviewed slides, while patients with colonoscopic tattooing who did not show such deposits were included among controls (N = 238) [7]. They detected a higher lymph node yield in patients with preoperative tattooing (N = 67), median 15 (IQR, 10–20) versus 12 (IQR, 9–16), (P < 0.014). In multivariable analysis, the presence of carbon-containing lymph nodes was an independent predictive factor for higher lymph node yield (P < 0.002) [7]. Unfortunately, due to the retrospective type of study, the Authors could not determine if the nodes were actually visibly black, which is relevant since preoperative colonoscopic tattooing could help identifying lymph nodes only if the ink is visible at inspection.

To assess the prevalence of microscopic ink deposits, we reviewed the slides of 30 out of 107 (28%) randomly selected tattooed patients. Only 2 out of 30 (7%) showed ink deposits, and we therefore decided not to examine the remaining tattooed patients. This finding is in accord with the feeling among both surgeons and pathologists who worked together in this study that macroscopic ink tattoo of lymph nodes is usually not detected at this institution in patients who underwent preoperative tattooing to mark the colonic site of the lesion rather than for sentinel lymph node biopsy technique.

## Conclusions

This study shows that endoscopic preoperative ink tattooing *to mark the site of the tumour* does not improve neither adequate lymphadenectomy nor lymph nodes yield in colorectal cancer specimen. Further studies are therefore needed to determine if colonoscopic tattooing can refine staging. Until then, endoscopic ink tattooing should be used if needed to mark the site of the tumour before surgical resection (e.g., small lesions, laparoscopic surgery), but not to increase the number of lymph nodes retrieved from specimen in patients with colorectal cancer.

## Methods

All patients who underwent an elective colonic or rectal resection for cancer between January 2009 and December 2011 at the S. Anna University Hospital in Ferrara (Italy) were identified retrospectively from a hospital discharge database. The operative reports of all patients were reviewed and patients who underwent an elective colorectal resection with regional lymphadenectomy for possible colorectal cancer were included in the study. Patients who underwent a segmental colectomy for a benign disease, a salvage resection for loco-regional recurrent colorectal cancer or a subtotal colectomy for synchronous tumours were excluded. The patients were then separated into two groups: patients in whom ink tattooing had been performed during a preoperative colonoscopy to mark the site of the tumour (tattoo group) and those in whom tattooing had not been performed (control group).

The choice whether to mark at preoperative colonoscopy the site of the tumour relied on the judgement of the endoscopist. At the preoperative colonoscopy, performed at a median of 23 days (IQR, 9.5-39 days) before the surgical operation, the tumour was localized and marked according to a standard protocol using black ink (S.A.L.F. S.p.a. Laboratorio Farmacologico Cenate Sotto, Bergamo, Italy). An injection needle was passed through the working channel of the endoscope and placed in the submucosa to deliver the ink: at first, a small deposit of saline solution was injected to verify the correct position, then the syringe was replaced and 0,5 to 1,0 mL of ink was delivered, followed by a new injection of 1,0 ml of saline solution [26]. If the lesion was located in the descending colon, rectosigmoid or ascending colon, tattoos were placed approximately 1 to 2 cm distal from the lesion and on two sides of the lumen, while for tumours located in the transverse colon they were placed both distally and proximally, and on both sides of the colon (i.e., at 3 and 9 o’clock).

The type of surgical resection was specified according to the vessels ligated by reviewing the operative reports.

The colorectal specimens were examined according to a standard procedure. Multiple tissue blocks of the tumour (about one for each cm of diameter) were submitted for examination. Pericolorectal adipose tissue was sliced every 5 mm and all macroscopically evident (palpable or visible) lymph nodes were manually dissected and microscopically examined. When a low number of lymph nodes was found the specimen was sampled again and if no more lymph nodes were retrieved, blocks of mesenteric fat were submitted for examination. The tumour was staged according to the TNM staging system [27].

The primary outcome measure was the number of adequate lymphadenectomies (defined as ≥12 lymph nodes) performed.

All factors known to influence the retrieval of lymph nodes were evaluated including patients’ age, gender, and body mass index (BMI), tumour location and size, TNM staging, DNA microsatellite instability-high (MSI-H) status, and preoperative chemo-radiation therapy status [10].

A random sample of patients in the tattoo group (N = 30) was selected for pathology slides review to detect presence of microscopic ink deposits, that was note usually described in the pathology reports.

The Unified Ethics Committee of the Province of Ferrara has granted an exemption from requiring ethics approval for this study. All patients signed a written informed consent. Data collection and analysis was performed in compliance with the Helsinki Declaration.

### Statistical analysis

Data are expressed as mean ± standard deviation or median (interquartile range – IQR 25–75) according to the distribution assessed by the Shapiro-Wilk test. Categorical data are presented as number (%). Data were analysed using the Chi-square, ANOVA, and Mann–Whitney tests to compare percentages, means, and non-parametric data, respectively. Logistic regression analysis was employed to construct a model predicting adequate lymphadenectomy considering factors regarding patient (age, gender, BMI, and preoperative colorectal tattooing), type of resection (colonic vs. rectal resection, right vs. left colonic resection), surgical technique (laparotomy vs. laparoscopy), surgeon’s experience (high- vs. low-volume), and pathologist (expertise in colorectal diseases vs. others). Significance was considered for P < 0.05. Statistical analysis was performed with IBM SPSS Statistics for Windows, Version 20.0 (IBM Corp. Released 2011. Armonk, NY: IBM Corp.).

## Abbreviations

TME: Total Mesorectal Excision
BMI: Body Mass Index
MSI-H: MicroSatellite Instability-High
